# How do we think and what is the neural circuit mechanism for it? Possible roles of working memory and inner speech in thinking

**DOI:** 10.3389/fnhum.2026.1722790

**Published:** 2026-06-03

**Authors:** Kensaku Mori, Hitoshi Sakano

**Affiliations:** 1 RIKEN Center for Brain Science, Saitama, Japan; 2 Department of Brain Function, School of Medical Sciences, University of Fukui, Fukui, Japan; 3 Department of Animal Behaviors, School of Veterinary Medicine, University of Tokyo, Tokyo, Japan

**Keywords:** cortical networks for thinking, cortico-cortical reverberatory circuits, cortico-thalamo-cortical loops, respiratory phases, self in the cognitive world, thinking with inner language, working memory

## Abstract

Animals detect changes in the surrounding situation and predict what is likely to happen next by evaluating the current sensory information to that of a prior associative memory. Using working memory with inner speech, humans are able to flexibly predict the future situation and devise appropriate strategies to avoid dangers or achieve goals. This prospective thinking is supported not only by feed-forward sensory information with associative memory but also by working memory that maintains attended information for tens of seconds. Working memory is a type of short-term memory essential for providing the temporal and spatial continuity of attention during the transition from the current behavior to the next. We hypothesize that repeated associative learning of sensory signals with multisensory object imagery may form the reverberatory circuits between the sensory cortices and higher cognitive areas. The cortico-cortical reverberatory circuits may generate the cognitive scenes of objects. We also hypothesize that the cortico-thalamo-cortical loops may maintain the cognitive object-scenes as working memory to internally search for related memories of action planning and emotional self-state. Such internal search for relevant memory engrams may be a major role of working memory in thinking. In this perspective article, we discuss self-cognition and the circuit mechanism for it in the human brain.

## Introduction

French philosopher René Descartes said, “I think, therefore I am”. Then, how do we think and what are we? These issues have long been discussed by neuroscientists, philosophers, and psychologists. Another question is whether there is a distinction between the animals and humans in cognizing the self. If so, what makes the difference between them in terms of circuit mechanisms for thinking? Animals detect changes in the surrounding situation through various sensory systems. Input information, when it is related to the survival of individuals and species, is evaluated by hard-wired innate circuits whose decisions are stereotyped as a result of natural selection (Kobayakawa et al., 2007; Mori and Sakano, 2021; Haimson and Mizrahi, 2025). Sensory information is also evaluated by the multi-synaptic learned circuits whose decisions are made based on the previous valence of the associative memory scene (Volz and von Cramon, 2009; Johansen et al., 2014; Namboodiri and Stuber, 2021).

Most animals make decisions quickly and rarely take long before responding to its environmental situation. In contrast, humans often take measures using working memory for further deliberation. Working memory is defined as a cognitive system that holds information temporarily (Goldman-Rakic, 1995; Chatham and Badre, 2015; Miller et al., 2018) for the organization of goal-directed behavior. It is a kind of short-term memory that allows for the manipulation and maintenance of stored information.

Recent evidence indicates that respiration-phase coherent neural activities occur in various cortical regions including prefrontal areas that are essential for making behavioral decisions (Ito et al., 2014; Chi et al., 2016; Biskamp et al., 2017; Herrero et al., 2018; Köszeghy et al., 2018; Moberly et al., 2018; Tort et al., 2018; Bagur et al., 2021; Girin et al., 2021; Kluger and Gross, 2021; Karalis and Sirota, 2022; Folschweiller and Sauer, 2023). Based on such accumulating evidence, we have previously proposed that in the rodent olfactory cortical system, one respiratory cycle may constitute a minimum time frame for one decision making (Mori and Sakano, 2024b, 2025a). In the orthonasal olfaction, sensory information is identified during the inhalation phase and then processed in the sensory cortex to recollect the associative memory scene for valence evaluation (Mori and Sakano, 2022a, 2022b, 2024a). During the following exhalation phase, decisions are made for behavioral and emotional outputs.

Humans use the learned language for thinking and recalling memories of words that represent cognitive scenes of objects, actions, and emotions. Inner speech may play a crucial role in deep and flexible thinking (Vygotsky, 1987; Andersen-Day et al., 2016; Fernyhough and Borghi, 2023). Although learned language was developed in humans to communicate with other individuals, it is also used for thinking without vocalization, hearing one’s own voice silently. Infants in their early stages use learned language for thinking by speaking aloud, however, they later learn to speak to themselves without verbal sounds during verbal thoughts (Vygotsky, 1987). Inner speech is helpful for pondering an issue, for example when we logically think of how to devise a strategy to solve a problem and achieve the goal. Mice usually detect olfactory information and make a behavioral output within a few respiratory cycles. Unlike mice, humans are able to continue thinking for deeper thought, blocking the behavioral output across many respiratory cycles to talk to themselves silently using inner speech (Fernyhough and Borghi, 2023). In this perspective article we will discuss the circuit mechanisms for thinking, focusing on the working memory and inner speech in the human brain.

### Cortico-cortical reverberatory circuits for generating cognitive scene signals

**Figure 1:**
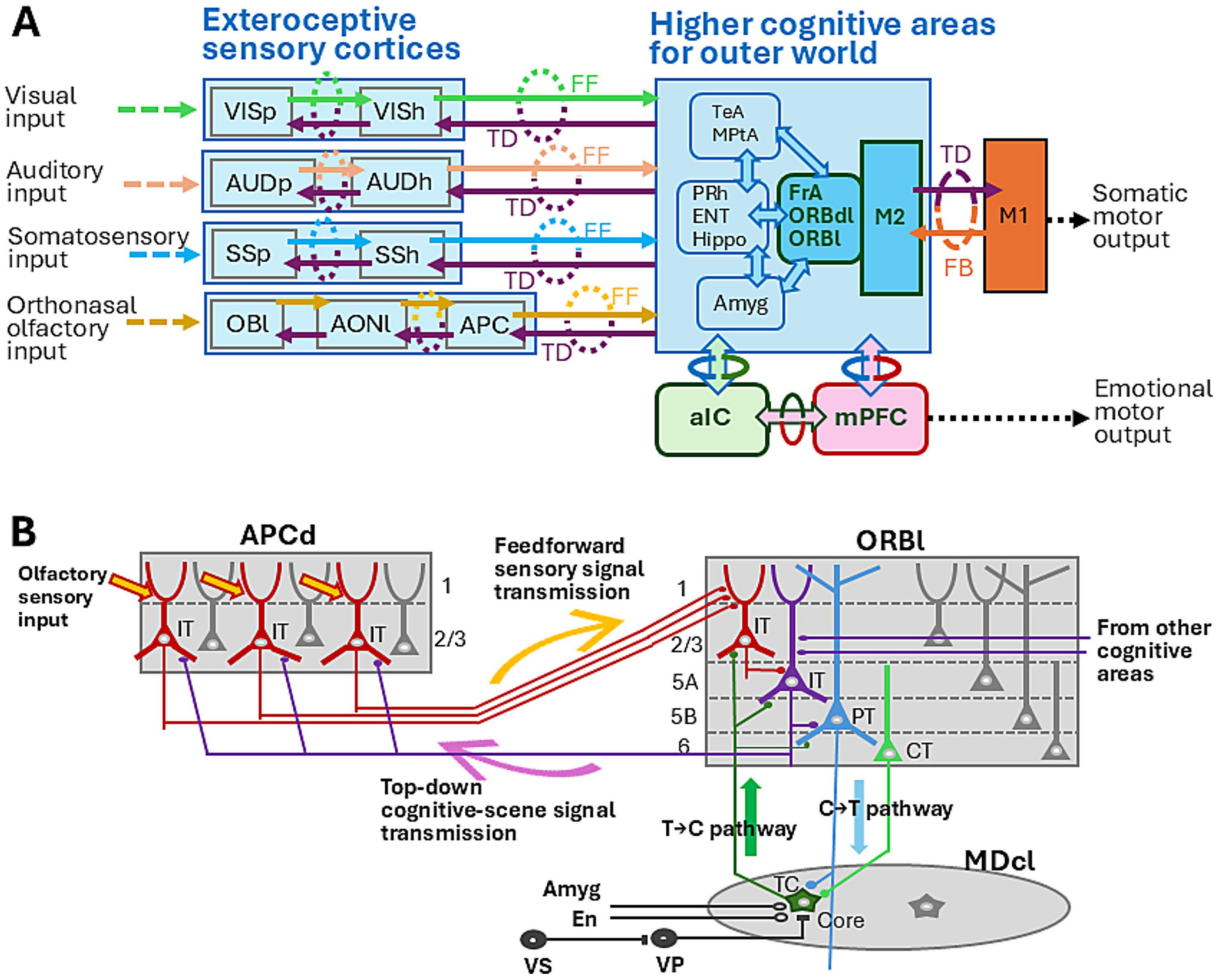
**(A)** Reverberatory circuits between the exteroceptive sensory cortices and higher cognitive areas for the outer world. Repeated associative learning of feedforward (FF) sensory object-signals with top-down (TD) cognitive scene signals may form the reverberatory circuits (shown by a pair of opposite-direction arrows with a dotted circle) between the sensory cortices and higher cognitive areas. Sustained firings in these reverberatory circuits may generate the cognitive scenes of objects. Repeated associative learning of TD cognitive scene signals of objects with feedback (FB) action planning signals may form the reverberatory circuits (shown by a pair of opposite-direction arrows with a broken circle) between the higher cortical areas and motor cortex. Sustained firings in the reverberatory circuits may give rise to action planning toward the object. Repeated associative learning of cognitive scenes of objects with the emotional state of the self may form the reverberatory circuits (a double-headed arrow with a solid circle) between the higher cognitive areas and medial prefrontal cortex (mPFC). Sustained firing activity in the reverberatory circuits may generate emotion toward the object. **(B)** A model of coupling between the cortico-cortical reverberatory circuits and cortico-thalamo-cortical loop. Repeated associative learning of odor object-signals with top-down cognitive scene signals may form the cortico-cortical reverberatory circuits between pyramidal cells in the dorsal subdivision of anterior piriform cortex (APCd) and pyramidal cells in the lateral subdivision of the orbitofrontal cortex (ORBl). In the reverberatory circuits, APCd pyramidal cells that are activated by the object odor (shown in dark red) send feedforward odor signals to pyramidal cells in the ORBl (a large yellow arrow) during the inhalation phase. In contrast, ORBl pyramidal cells that generate cognitive scene signals transmit the signals back to pyramidal cells in the APCd (a large purple arrow). Sustained firings of these pyramidal cells may participate in generating working memory of the olfactory cognitive percepts. Pyramidal-tract neurons in layer 5 (PT) and cortico-thalamic neurons in layer 6 (CT) of the ORBl project axons to thalamo-cortical neurons (TC) in the centrolateral subdivision of the thalamic mediodorsal nucleus (MDcl). The core type TC cells (Jones, 2007) send axons mainly to intratelencephalic (IT) neurons in the ORBl. Then, IT neurons send signals to PT and TC neurons, thus forming the cortico-thalamo-cortical loop. This cortico-thalamo-cortical loop may be responsible for maintaining working memory of the cognitive scenes of objects. Classification of cortical pyramidal cells into IT, PT, and CT neurons is from Shepherd and Yamawaki (2021). TC neurons in the MDcl receive excitatory inputs from the amygdala (Amyg) and endopririform nucleus (En), and inhibitory inputs from the ventral pallidum (VP). See the glossary for abbreviations.

Thinking starts with generating the cognitive percepts of objects and internal state of ourselves. What is the neural circuit mechanisms for the generation of cognitive percepts? We previously proposed a model of respiration-phase coherent interactions between the olfactory cortex areas and higher cognitive areas (Mori and Sakano, 2025a) based on the current source-density analysis of local field potentials in the olfactory cortex (Narikiyo et al., 2018). We hypothesized that during the inhalation phase, feedforward odor signals of an external object drive burst firings of a specific subset of pyramidal cells in the olfactory cortex. We assumed that, during the subsequent late-exhalation phase, the higher cognitive areas generate cognitive scene signals of the object and transmit them via the top-down pathway back to the olfactory cortex. We also hypothesized that the top-down cognitive signals activate the same subset of pyramidal cells as that activated by the feedforward odor signals (Figures 1A,B). The term “cognitive scene of object” refers to the neural network activity that is generated by higher cognitive areas in response to the multiple different sensory inputs from the object (Figure 1A). We assume that the network activity may represent multisensory-integrated percepts of the object. It should be emphasized that the higher cognitive areas generate the “cognitive scene signals” only after the establishment of associative learning of sensory inputs with multisensory percepts of the object.

At the early stage of odor-object associative learning, odor-signals alone without other sensory input-signals may not be able to generate cognitive scene-signals of an object in the higher cognitive areas. However, after establishing odor-object associative memory by repeatedly experiencing multisensory inputs from the object, odor-induced activity of olfactory-cortex pyramidal cells can activate the cognitive scene-signals in higher cortical areas. The odor-induced cognitive scene-signals may be transmitted back to the same pyramidal cells in the olfactory cortex and reactivate them. In other words, reverberatory circuits between the olfactory cortex areas and higher cognitive areas may be formed by the reciprocal synaptic connections of object-odor tuned pyramidal cells in the olfactory cortex with pyramidal cells in the higher areas that generate the cognitive scene-signals of the object (Figure 1B). Thus, after the establishment of odor-object associative learning, olfactory inputs during the inhalation phase can activate the reverberatory circuits resulting in the generation of olfactory cognitive percepts. We hypothesize that one respiratory cycle provides a fundamental time unit for generating one olfactory cognitive percept of an external object.

We further speculate that the top-down cognitive scene signals are transmitted back not only to the olfactory cortex but also to the visual, auditory, and somatosensory cortices during the late-exhalation phase (Figure 1A) (Mori and Sakano, 2025a). Thus, reverberatory circuits may be formed between the visual cortex and higher areas, generating visual cognitive percepts of an object. By the same token, the reverberatory circuits between the auditory cortex and higher areas may generate auditory cognitive percepts, while those between the somatosensory cortex and higher areas may be responsible for generating somatosensory cognitive percepts. Finally, simultaneous activation of these reverberatory circuits between the sensory cortices and higher areas may result in the multisensory object-percepts, i.e., a coherent representation of the object combining the olfactory, visual, auditory, and somatosensory percepts. We thus hypothesize that sensory inputs from an object may result in the generation of multisensory cognitive percepts in these reverberatory circuits during the late-exhalation phase. We hypothesize that one respiratory cycle provides a basic time frame for the integration of “feedforward sensory information” and “top-down cognitive scene information” for generating multisensory object-percepts.

It has been reported that in the sensory or cognitive tasks that require attention, humans actively adjust the inhalation phase to stimulus presentation and the exhalation phase to their responses or decisions (Melnychuk et al., 2018; Perl et al., 2019; Johannknecht and Kayser, 2022; Grund et al., 2022; Andrews et al., 2025). In addition, humans consciously adjust the respiratory phases to vocalization such as singing and speaking (Correia et al., 2020). These studies suggest that neocortically-instructed active/intentional respiration may influence a wide variety of cortical functions in humans. It should be noted, however, that autonomic respiration without neocortical instruction may not influence some cortical functions (Thunell et al., 2024). We previously proposed the hypothesis that in the rodent brain, one intentional respiratory cycle provides a basic time frame for cognition and decision-making (Mori and Sakano, 2024b, 2025a). Based on these studies in humans, we speculate that the above hypothesis may be applicable to the human brain.

Cortical networks in mammals often transform the multisensory percepts of an object into actions toward the object such as pursuing prey or hiding from predators. To adequately perform these actions, it is necessary to maintain the cognitive percepts for tens of seconds (beyond one respiratory cycle) to search for and recall stored memories of actions toward the object. What is the neural circuit mechanism for maintaining cognitive percepts? We speculate that the networks of higher cognitive areas and higher-order thalamus, such as the thalamic mediodorsal nucleus (MD), may maintain the cortico-cortical reverberatory activity that represents the cognitive percepts as working memory (Goldman-Rakic, 1995; Miller et al., 2018; Parnaudeau et al., 2018).

Working memory can be classified into three categories. The first type is “working memory of cognitive scenes of objects” as described above. We hypothesize that the reverberatory circuits between sensory cortices and higher cognitive areas may generate cognitive percepts and that cortico-thalamo-cortical loops between higher cognitive areas and higher thalamus may be responsible for maintaining the cognitive percepts as working memory (Figure 1A). The second type is “working memory of action planning” that maintains and works with motor preparation (Svoboda and Li, 2018). Repeated associative learning of the cognitive scene with specific action planning toward the object may form reverberatory circuits between higher cognitive areas and the motor cortex (Figure 1A). We speculate that these reverberatory circuits may be responsible for generating action plans (Svoboda and Li, 2018; Yang and Kwan, 2021; Chen et al., 2024). We further assume that the cortico-thalamo-cortical loops between the higher cognitive areas (including the secondary motor cortex M2) and higher thalamus maintain the action planning as working memory. The third type is “working memory of emotional states of the self” or “affective working memory” that maintains and works with emotional states (Barrett et al., 2007; Baddeley, 2013; Mikels and Reuter-Lorents, 2019) (Figure 1A). Repeated associative learning of the cognitive scene of an object with the specific emotional states of the self may form the reverberatory circuits between the higher cognitive areas and medial prefrontal cortex (mPFC) (Figure 1A). We hypothesize that these reverberatory circuits may generate emotional scenes associated with the objects and that the cortico-thalamo-cortical loops between the mPFC and higher thalamus may maintain the emotional scenes as working memory.

### Cortico-thalamo-cortical loops for maintaining working memory

**Figure 2:**
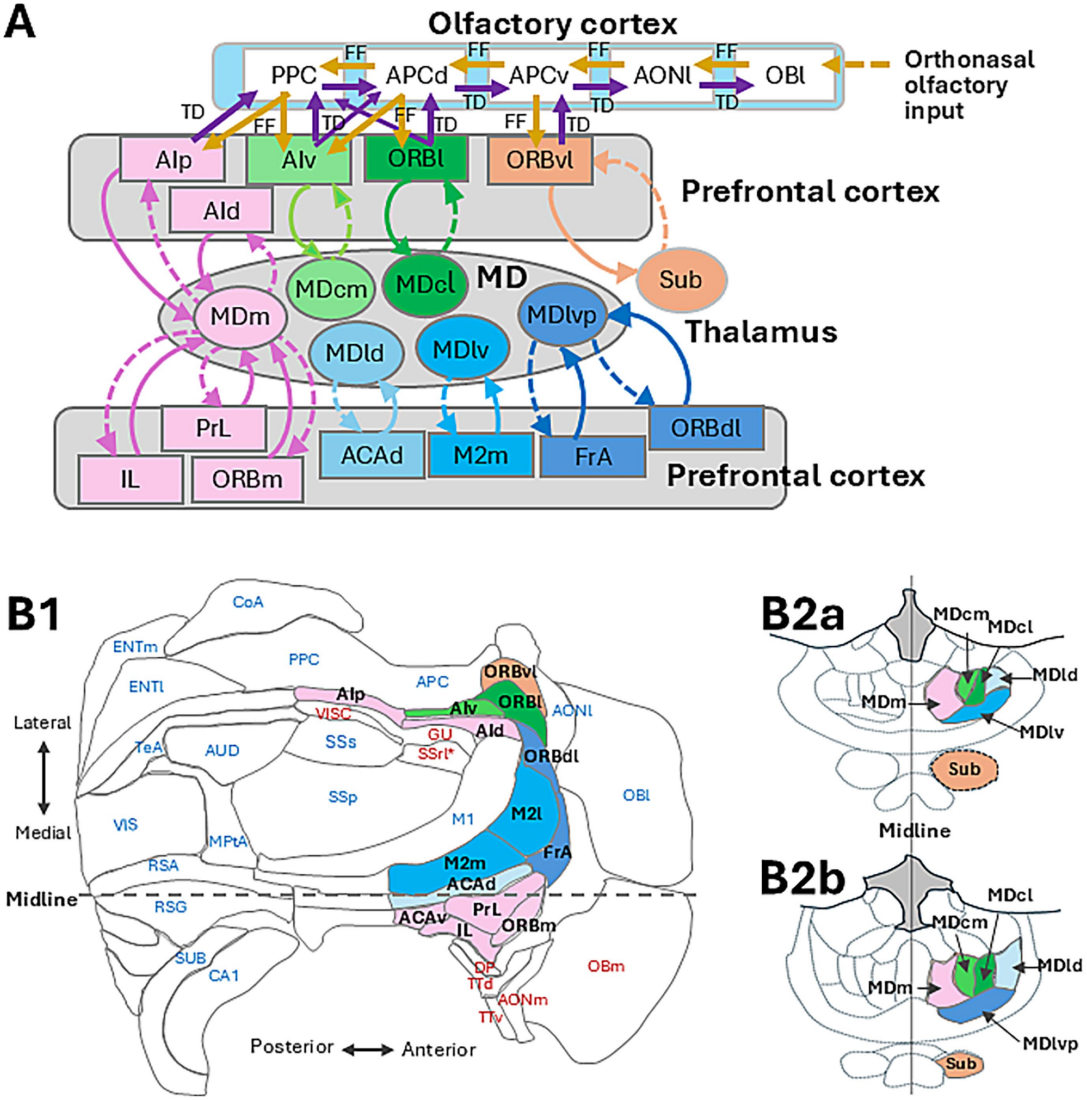
**(A)** Diagrams showing that different areas of the prefrontal cortex (PFC) form the cortico-thalamo-cortical loops with distinct subregions of the thalamic mediodorsal nucleus (MD). These loops include ORBl⇌MDcl (shown in dark green), AIv⇌MDcm (pale green), ORBdl⇌MDlvp (dark blue), FrA⇌MDlvp (dark blue), M2m⇌MDlv (blue), ACAd⇌MDld (pale blue), AIp⇌MDm (pink), AId⇌MDm (pink), PrL⇌MDm (pink), IL⇌MDm (pink), and ORBm⇌MDm (pink). ORBvl forms the cortico-thalamo-cortical loops with the thalamic submedial nucleus (Sub). This diagram also illustrates how the olfactory cortical areas connect to the prefrontal cortex areas. See the glossary for abbreviations. **(B)** Spatial arrangements of prefrontal cortex areas in the unfolded map of the mouse cerebral hemisphere (dorsal view) (B1) and subregions of the mediodorsal nucleus (MD) in the coronal sections through the MD (B2). B1a and B1b illustrate the rostral and caudal sections, respectively. B1 and B2 are modified from Paxinos and Franklin (2001). Colors in the PFC areas and MD subregions in **(B)** correspond to those in **(A)**. Adopted from Mori and Sakano (2022a).

In rodents, the prefrontal cortical (PFC) areas reciprocally connect with the higher-order thalamic nuclei including the mediodorsal nucleus (MD) (Figures 1B, 2) (Krettek and Price, 1977; Ray and Price, 1992; Jones, 2007; Collins et al., 2018; Shepherd and Yamawaki, 2021). It has been demonstrated that the cortico-thalamo-cortical loops in the PFC network play a key role in maintaining cognitive percepts and planning actions as working memory (Bolkan et al., 2017; Guo et al., 2017; Schmitt et al., 2017). As illustrated in Figure 2A, each area of the rat PFC reciprocally connects with distinct subregions of the thalamic mediodorsal nucleus (MD) (Ray and Price, 1992). The lateral orbitofrontal cortex (ORBl), for example, reciprocally connects with the centrolateral subregion of MD (MDcl), forming the cortico-thalamo-cortical (ORBl⇌MDcl) loops. The ORBl and the dorsal subregion of the anterior piriform cortex (APCd) may form the cortico-cortical (APCd⇌ORBl) reverberatory circuits because the ORBl receives feedforward olfactory sensory inputs from the APCd and sends top-down axons back to the APCd (Neville and Haberly, 2004; Illig, 2005; Chen et al., 2014) (Figure 1B). This raises the possibility that the ORBl⇌MDcl loops may be functionally coupled with the APCd⇌ORBl reverberatory circuits.

Figure 1B illustrates a possible model of the neural circuit that links the cortico-cortical reverberatory circuits with the cortico-thalamo-cortical loops. We speculate that intratelencephalic (IT) neurons in the APCd may send feedforward sensory signals to IT neurons in the ORBl, and that IT neurons in the ORBl send top-down cognitive scene signals back to IT neurons in the APCd. Thus, the cortico-cortical reverberatory circuits may mostly be composed of IT neurons. In contrast, the cortico-thalamo-cortical loops include not only IT neurons but also pyramidal tract (PT) neurons and corticothalamic (CT) neurons in the ORBl (Anastasiades and Carter, 2021; Shepherd and Yamawaki, 2021). PT neurons in layer 5B project axons to subcerebral targets and send axon-collaterals to core-type thalamocortical (TC) neurons in the MDcl (Jones, 2007). In addition, CT neurons in layer 6 project axons to TC neurons. Thus, the cortico-thalamic pathway consists of PT and CT neurons excluding IT neurons (a blue arrow in Figure 1B). TC neurons in the MDcl send axons densely to IT neurons but weakly to PT and TC neurons in the ORBl, forming the thalamo-cortical pathway (a green arrow in Figure 1B). Finaly, IT neurons may send signals to PT neurons and CT neurons in the ORBl (Kawaguchi, 2017). We therefore speculate that the cortico-thalamo-cortical loops of the ORBl consist of IT→PT/CT → TC → IT excitatory connections.

With these considerations in mind, we assume that synaptic connections from IT neurons to PT and TC neurons play a key role in functionally coupling the cortico-cortical reverberatory circuits with the cortico-thalamo-cortical loops in the APCd-ORBl-MDcl networks. We further speculate that the APCd⇌ORBl cortico-cortical reverberatory circuits may participate in generating the cognitive object-percepts and that the ORBl⇌MDcl cortico-thalamo-cortical loops maintain the cognitive percepts for a short period of time as working memory. Contents of the olfactory cognitive scenes may be coded in the specific reverberatory circuits between the APCd and ORBl, whereas the ORBl⇌MDcl cortico-thalamo-cortical loops may determine whether the olfactory cognitive scenes are maintained as working memory or not.

The ventral agranular insular cortex (AIv) reciprocally connects with the APCd and the posterior piriform cortex (PPC) (Neville and Haberly, 2004; Chen et al., 2014) (Figure 2A). We thus speculate that the APCd/PPC and AIv form the cortico-cortical (APCd/PPC⇌AIv) reverberatory circuits. In addition, the AIv reciprocally connects with the centromedial subregion of the thalamic mediodorsal nucleus (MDcm). This raises the possibility that neural circuits in the AIv provide a node for coupling of the APCd/PPC⇌AIv cortico-cortical circuits with the AIv⇌MDcm cortico-thalamo-cortical loops. We hypothesize that the APCd/PPC⇌AIv⇌MDcm networks may also be involved in generating the olfactory cognitive scenes and its short-term maintenance as working memory.

As illustrated in Figure 1A, the secondary motor cortex (M2) has reciprocal connections with the primary motor cortex (M1) which form the cortico-cortical (M2⇌M1) reverberatory circuits that may generate action planning signals (Barthas and Kwan, 2017; Svoboda and Li, 2018). The medial part of the M2 (M2m in Figure 2) has reciprocal connections with the lateroventral subregion of the thalamic mediodorsal nucleus (MDlv), suggesting that the M2m and MDlv may form the cortico-thalamo-cortical loops for maintaining the action planning signals as working memory (Yang and Kwan, 2021; Chen et al., 2024).

### Working memory for thinking in the imaginary world

In the previous sections, we discussed circuit mechanisms of thinking along with working memory for the outer world. Thinking also occurs for the inner world, not only for the inner sensory information but also for the recalled associative-memory scene (Mildner and Tamir, 2019; Mori and Sakano, 2025b). Thus, the inner cognitive world appears to be generated by interoceptive sensory stimuli and activation of memory engrams to evaluate the current self-status. As seen for the exteroceptive information from the outer world, working memory may also play an important role in thinking about the inner world so that the person can respond to the situation within the body, solve the problem, and achieve the goal. Humans evaluate the current status of self in the cognitive world and try to make the situation more comfortable. Usually, we do not keep an inner thought-object too long and flexibly change it while thinking. Humans are able to volitionally manipulate working memory in the inner imaginary world without being dominated by the inputs from the outer world, so that they can choose what to think about and decide whether to take action (Miller et al., 2018).

Compared with other animals, humans appear to evaluate the surrounding situation by observing ourselves more objectively in the environmental scene. In the inner cognitive world, we find the self in the imaginary scene that can be induced by sensory signals and by spontaneous firing of memory engrams. When we find the third-person self in the cognitive world, we evaluate the current status, whether it is satisfactory or not. We are able to locate ourselves not only in the outer world, but also in the cognitive space in the inner world by estimating the distance to the goal (an ideal situation) and from the crisis (potential danger) to avoid. This self-locating mechanism in the imaginary world may have evolved from the self-navigation mechanism (Nyberg et al., 2022) that is commonly found in other animals for the real outer world, when searching for food or escaping from predators. Thus, objective cognition of self appears to be important for thinking about oneself in both the real world and imaginary world.

Thinking may be classified into two different categories. One is passive thinking, which is driven by sensory input from the outer world to respond to the changes in the environment in a timely manner. The other is active thinking about the self, which is self-driven to evaluate the current situation of self in the imaginary world. This self-driven thinking about the self occurs when we are relaxed without being disturbed by sensory information from the outer world. In either way of thinking, world-driven or self-driven, we take measures to improve the situation and devise a strategy to achieve this goal. After evaluating the self-situation by thinking, either passively or actively, neural circuit mechanisms of formulating the emotional and behavioral outputs appear to be the same for both outer and inner worlds.

### Thinking process internally associates cognitive scenes with action planning

To perform goal-directed behaviors, the mammalian cerebral cortex internally searches for and recall memories of not only the goal percepts but also the cognitive scenes of task-relevant events in the outer world. Let us consider a hungry mouse thinking about eating food. The mouse’s thinking may start with imagining a variety of possible foods, i.e., internally searching for and recalling memories of learned foods. It is not known yet how the brain internally generates the cognitive percepts of foods without sensory inputs. One possibility is that the cortico-thalamo-cortical loops may be able to activate the reverberatory circuits between the higher cognitive areas and sensory cortices (Figure 1B), thus internally generating the cognitive scenes of foods. The initial thinking-cycle ends with the choice of a specific food among the recalled candidate foods. Once the mouse comes up with a particular food such as wheat grains, it then searches for memories of possible learned pathways leading to the place where the intended food might be found. By maintaining the cognitive scenes of specific food as working memory, cortical networks of the hungry mouse can internally search and recall memories of a series of cognitive scenes that were experienced along the pathway from the nest to the place where the food was present. Such cognitive scenes of the pathway may be encoded by reverberatory circuits between the visual / somatosensory cortices and higher areas for spatial cognition. We hypothesize that in the second thinking cycle, the mouse maintains the recalled cognitive scenes of the pathway as working memory for navigation to its goal. Thus, thinking appears to consist of a chain of working memory cycles.

Through the process of maintaining working memory, the cortical networks can recall memories of action plannings toward the objects. This leads to the generation of action-planning signals in the reverberatory circuits between the higher cognitive areas and motor cortex (a broken-line circle in Figure 1A) (Chen et al., 2024). By maintaining the action preparation as working memory, the cortex can quickly execute the planned action whenever the appropriate situation occurs. If a mouse finds a peanut, the mouse may plan to eat it or flexibly change the plan to stuff it in the cheek pouch and bring the peanut to the nest if a rival mouse appears. After executing the action toward the objects, the cortical networks detect sensory information of the updated external world (such as no food anymore) and may start responding to the new situation. We hypothesize that one basic cycle of thinking may be composed of these chains of cortical processes linking the cognitive object-scenes with appropriate action plannings toward the objects.

We also assume that by means of maintaining cognitive scenes of a specific object as working memory, the cortical networks may search for stored memories of the emotional state of the self that were learned in association with the object. Thus, the generation of working memory of a specific object may induce internal searching for a variety of stored memories of both relevant objects and associated emotion of the self. Such internal search for relevant memories would form the basis of the thinking process.

### Possible neural circuits for volitional control of cognitive scenes

As discussed above, we propose the model of functional coupling between the cortico-cortical reverberatory circuits with the cortico-thalamo (MD)-cortical loops (Figures 1B, 2A). We hypothesize that couplings of the cortico-cortical reverberatory circuits with the cortico-thalamo-cortical loops may play a key role in maintaining the generated cognitive scenes as working memory. Humans are able to volitionally control the generation of cognitive scenes and intentionally maintain them as working memory (Miller et al., 2018). What are the neural circuit mechanisms for these processes? It is possible that in the reverberatory circuits of sensory cortex-PFC, PFC nodes receive the lateral control inputs from other higher cognitive areas (Figure 1B). These inputs may activate PFC pyramidal cells that represent cognitive object-percepts, resulting in internal percept generation. Another possibility is that the forebrain internally controls the activity of PFC⇌MD loops by modulating MD-neuron activity. For example, MDm and MDc receive the excitatory inputs from the amygdala and endopririform nucleus, as well as the inhibitory inputs from the ventral pallidum (Figure 1B) (Kuroda and Price, 1991; Kuroda et al., 1998). This may suggest that these inputs internally control the activity of PFC⇌MD loops and thus, the maintenance and termination of working memory. By maintaining the cognitive scene of a specific goal as working memory, the cortical networks internally search for and recollect long-term memories of objects that are related to the goal. Furthermore, by maintaining the cognitive object-scenes, the cortical networks also search for and recall memories of emotions and action planning of the self toward the object. In other words, working memory may be a cortical network mechanism for internally searching and recollecting long-term memories of other things that are related to the object. In the goal-directed behaviors of animals and humans, a basic cycle of thinking consists of internal search for long-term memories of goal-related episodes and internal recall of memories that may be useful in attaining the goal. We speculate that thinking develops by way of advancing the chains of these basic cycles toward the goal.

## Discussion

“What are we?” This question is commonly asked when the self is recognized in the imaginary world. “I think, therefore I am”. This may be the answer by the philosopher, but neuroscientists must be able to describe the thinking and self-cognition processes in terms of neural circuits. Thinking may be defined as a cognitive process that happens internally and independently of sensory stimuli in the imaginary world. Thinking includes judging the situation, reasoning the strategy, solving the problem, considering the idea, and imaging the future. In humans, thinking may be a form of inner speech in which words are silently expressed in the mind. Inner speech is self-talk using inner language, thinking to oneself, and is intra-personal verbal but silent communication (Vygotsky, 1987; Fernyhough and Borghi, 2023). Inner speech is used for planning, reasoning, dreaming, introspection, and self-persuasion. How is inner speech related to our imaginary thoughts? Inner speech appears to direct our attention and motivation to achieve the goal and play an important role in thinking to devise the strategy (Alderson-Day et al., 2016; Fernyhough and Borghi, 2023).

Because respiration-phase coherent neuronal activities have been reported in various cortical regions including the prefrontal areas that are essential for decision-making (Ito et al., 2014; Chi et al., 2016; Biskamp et al., 2017; Herrero et al., 2018; Köszeghy et al., 2018; Moberly et al., 2018; Tort et al., 2018; Bagur et al., 2021; Girin et al., 2021; Kluger and Gross, 2021; Karalis and Sirota, 2022; Folschweiller and Sauer, 2023), we speculate that animals including humans may make one decision during one respiratory cycle. When animals need to know more about a situation, they attempt to collect additional information, keeping the present sensory information in the working memory for the following respiratory cycles. Working memory may recall the related memory scenes for deeper thinking. Humans analyze the situation by thinking for further consideration, holding the behavioral and emotional responses in the exhalation phase. Why are we able to logically think in the imaginary world by looking at the situation and the self? Inner speech with learned language appears to play an important role in thinking (Fernyhough and Borghi, 2023). We can make abstract thinking by using the learned words that represent the categorical imageries of the scene, situation, and ideas. Then, how do we think about the subject in the imaginary world using working memory with inner speech, keeping our attention on the object in the real world? How can we abstract the situation into the inner words to logically think about and solve the problem? This abstraction and thinking processes need to be further clarified in the future at the level of neural circuits.

We propose that generation of cognitive scenes and maintenance of them as working memory in the forebrain may provide a neural basis for thinking. The learned language is a primary means for thinking about oneself and communicating with others. In vocal communication, volitional exhalation plays a key role in instructing the timing of speaking. Humans may acquire the ability to think about the external event, internal emotions, and action planning using the inner language. Learned words in human babies are initially simple such as “mom” and “dad.” However, as they grow, they accumulate the knowledge of words to speak and understand the complex sentences consisting of a specific sequence of multiple words. Furthermore, humans appear to acquire the ability to logically think using their inner language. For thinking, we learn to use not only complex words but also other characters, e.g., mathematical symbols and musical notes. First learned mathematical symbols are simple numbers such as 1, 2, 3, and basic operations, i.e., + for addition and − for subtraction. However, by accumulating and expanding this knowledge, we become able to work on complex mathematics such as differential calculus and determinant calculations. Therefore, studies on the human ability to think using words and symbols may help us understand a neural-circuit basis for various cultural activities including the sciences. We assume that thinking about an external object with inner speech recall associative behavioral actions in the related memory scenes. In addition, thinking about the self as an object in the cognitive world may also recall the emotional states in the scene. This thinking may result in the association of self-imagery with specific inner words that represent our feeling and mood, such as glad, angry, sad, and happy. Thus, elucidating the forebrain mechanisms for thinking may provide us with a neural basis of writing novels and scripts in describing the characters.

It has been proposed that a key function of remembering the past is to provide memory scenes to predict the future, for example what is likely to happen next, via imagined scenarios (Schacter et al., 2012). We speculate that a major role in recalling memory events and maintaining the recalled information as working memory is to internally probe the memory engrams for action planning toward the future event, predicting what will happen after the action. Regarding the time dimension, thinking is classified into three types; about the present, past, and future (Shipp and Aeon, 2019; Beaty et al., 2019). First, thinking about the present appears to be a sensory-driven process that involves recalling the imagery of external and internal states and maintaining them as working memory to develop a strategy for immediate action. Thinking about the present usually occurs in a few respiratory cycles. Through the gradual accumulation of learned memories, animals as well as humans become able to operate the present thinking via working memory (Hoerl and McCormack, 2018). Second, thinking about the distant past may involve the processes of recalling long-term memories in the past and searching for the related memory events, maintaining them as working memory. Retrospective thinking by looking back at stored memory of events is highly developed in human adults as the ability to internally drive the mental time-travel (Beaty et al., 2019) to recognize the causal relationship of two or more associated events (Hoerl and McCormack, 2018). Third, thinking about the distant future may also involve the internally driven processes of recalling memories to think how events will develop, keeping them as working memory for future planning. Prospective thinking about the distant future, i.e., predicting what is likely to happen, appears to be particularly evident in humans, which is known as a type of mental time-travel such as future-oriented self-projection (Irish et al., 2012; Beaty et al., 2019). We hypothesize that generating cognitive percepts and holding them as working memory may be the network basis of our thinking about the present, past, and future events. It is not well understood yet how our motivation and attention to the goal may affect the thinking process. Ventral and medial areas of the PFC including the anterior insular cortex (aI) are known to regulate motivated behaviors, attention, and emotional responses (Friedman and Robbins, 2022; Howland et al., 2022; Rolls, 2023; Soares-Cunha and Heinsbroek, 2023). These areas project to the ventral striatum and form the ventral cortico-striatopallido-thalamo-cortical loops (Ongür and Price, 2000; Ikemoto, 2010; Root et al., 2015; Haber, 2016; Anastasiades and Carter, 2021; Soares-Cunha and Heinsbroek, 2023). We speculate that the ventral cortico-striatopallido-thalamo-cortical loops interact with the cortico-thalamo (MD)-cortical loops and cortico-cortical reverberatory circuits. Future studies of these interactions will provide clues for our understanding of the network mechanisms for the effects of motivation, attention, and emotion in the thinking processes.

As discussed above, thinking appears to play an important role in remembering the past and predicting the future. During these processes, we recognize ourselves in imaginary worlds. Thinking often makes us fantasize about the past, present and future, and ponder “Where do we come from, what are we, and where are we going?” as asked by Paul Gauguin. This type of thinking sometimes leads us to the imaginary world causing us anxiety. However, thinking may also be a strong tool in positively evaluating the current situation, predicting the future, and recalling the past. As higher brain function appears to have evolved as a result of natural selection, it can be and must be used positively for the survival of individuals and species. It is hoped that understanding ourselves in terms of neuroscience at the level of neural circuits will make us (humans) wise and set us (individuals) mentally free.

## Data Availability

The original contributions presented in the study are included in the article/supplementary material, further inquiries can be directed to the corresponding authors.

## Glossary

ACAd: dorsal part of the anterior cingulate cortex
ACAv: ventral part of the anterior cingulate cortex
AId: dorsal agranular insular cortex
AIv: ventral agranular insular cortex
AIp: posterior agranular insular cortex
AONm: medial part of the anterior olfactory nucleus
AONl: lateral part of the anterior olfactory nucleus
APC: anterior piriform cortex
APCd: dorsal subregion of the anterior piriform cortex
APCv: ventral subregion of the anterior piriform cortex
AUDh: higher auditory cortex
AUDp: primary auditory cortex
Amyg: amygdala
aIC: anterior insular cortex
CA1: field CA1 of the hippocampus
CoA: cortical nucleus of amygdala
CT: corticothalamic neuron
C→T pathway: cortico-thalamic pathway
DP: dorsal peduncular cortex
ENT: entorhinal cortex
ENTl: lateral entorhinal cortex
ENTm: medial entorhinal cortex
En: endopiriform nucleus
FB: feedback transmission
FF: feedforward transmission
FrA: frontal association cortex
Gu: gustatory cortex
Hippo: hippocampus
IL: infralimbic cortex
IT: intratelencephalic neuron
MD: thalamic mediodorsal nucleus
MDc: central subregion of the thalamic mediodorsal nucleus
MDcl: centrolateral subregion of the thalamic mediodorsal nucleus
MDcm: centromedial subregion of the thalamic mediodorsal nucleus
MDld: laterodorsal subregion of the thalamic mediodorsal nucleus
MDlv: lateroventral subregion of the thalamic mediodorsal nucleus
MDlvp: lateroventroposterior subregion of the thalamic mediodorsal nucleus
MDm: medial subregion of the thalamic mediodorsal nucleus
MPtA: Medial parietal association cortex
M1: primary motor cortex
M2: secondary motor cortex
M2l: lateral part of the secondary motor cortex
M2m: medial part of the secondary motor cortex
mPFC: medial prefrontal cortex
OBl: lateral map of the olfactory bulb
OBm: medial map of the olfactory bulb
ORBdl: dorsolateral orbitofrontal cortex
ORBl: lateral orbitofrontal cortex
ORBm: medial orbitofrontal cortex
ORBvl: ventrolateral orbitofrontal cortex
PFC: prefrontal cortex
PPC: posterior piriform cortex
PRh: perirhinal cortex
PT: pyramidal tract neuron
PrL: prelimbic cortex
RSA: retrosplenial agranular cortex
RSG: retrosplenial granular cortex
SSh: higher somatosensory cortex
SSp: primary somatosensory cortex
SSrl*: rostrolateral part of the somatosensory cortex
SSs: secondary somatosensory cortex
SUB: subiculum
Sub: submedial nucleus of the thalamus
TC: thalamocortical neuron
T → C pathway: thalamo-cortical pathway
TD: top-down transmission
TTd: dorsal tenia tecta
TTv: ventral tenia tecta
TeA: temporal association cortex
VISh: higher visual cortex
VISp: primary visual cortex
VISC: visceral cortex
VP: ventral pallidum
VS: ventral striatum
